# (1*H*-1,2,3-Benzotriazol-1-yl)methyl benzoate

**DOI:** 10.1107/S1600536812015140

**Published:** 2012-04-18

**Authors:** Ting Guo, Gang Cao, Sen Xu

**Affiliations:** a Department of Stomatology, Nanjing Jinling Hospital, Nanjing University School of Medicine, 305, East Zhongshan Road, 210002 Nanjing, Jiangsu Province, People’s Republic of China; b Department of Applied Chemistry, School of Material Science and Engineering, Nanjing University of Aeronautics and Astronautics, Nanjing, Jiangsu Province 210016, People’s Republic of China

## Abstract

In the title compound, C_14_H_11_N_3_O_2_, the dihedral angle between the phenyl ring and the benzotriazole ring system is 76.80 (19)° and the mol­ecule has an L-shaped conformation. In the crystal, weak aromatic π–π stacking is observed, the closest centroid–centroid distance being 3.754 (2) Å.

## Related literature

For related structures and the synthesis, see: Xu & Shen (2012) ▶; Zeng & Jian (2009 ▶). For applications of benzotriazole derivatives, see: Wan & Lv (2010 ▶).
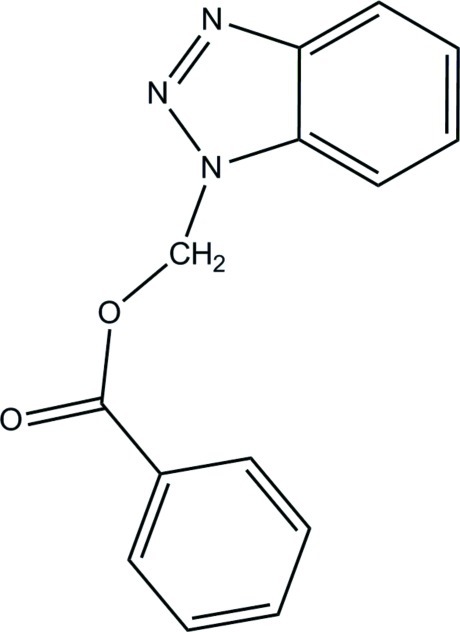


## Experimental

### 

#### Crystal data

C_14_H_11_N_3_O_2_
*M* *_r_* = 253.26
Monoclinic, 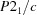

*a* = 10.7181 (4) Å
*b* = 6.4826 (2) Å
*c* = 18.7076 (7) Å
β = 96.773 (3)°
*V* = 1290.75 (8) Å^3^
*Z* = 4
Mo *K*α radiation
μ = 0.09 mm^−1^
*T* = 296 K
0.20 × 0.20 × 0.18 mm

#### Data collection

Bruker SMART CCD diffractometer
Absorption correction: multi-scan (*SADABS*; Bruker, 2001 ▶) *T*_min_ = 0.982, *T*_max_ = 0.984
9412 measured reflections
2268 independent reflections
1352 reflections with *I* > 2σ(*I*)
*R*_int_ = 0.057

#### Refinement

*R*[*F* ^2^ > 2σ(*F* ^2^)] = 0.060
*wR*(*F* ^2^) = 0.162
*S* = 1.06
2268 reflections
181 parameters
H atoms treated by a mixture of independent and constrained refinement
Δρ_max_ = 0.14 e Å^−3^
Δρ_min_ = −0.17 e Å^−3^

### 

Data collection: *SMART* (Bruker, 2007 ▶); cell refinement: *SAINT* (Bruker, 2007 ▶); data reduction: *SAINT*; program(s) used to solve structure: *SHELXS97* (Sheldrick, 2008 ▶); program(s) used to refine structure: *SHELXL97* (Sheldrick, 2008 ▶); molecular graphics: *DIAMOND* (Brandenburg, 1999 ▶); software used to prepare material for publication: *SHELXTL* (Sheldrick, 2008 ▶).

## Supplementary Material

Crystal structure: contains datablock(s) global, I. DOI: 10.1107/S1600536812015140/hb6707sup1.cif

Structure factors: contains datablock(s) I. DOI: 10.1107/S1600536812015140/hb6707Isup2.hkl

Supplementary material file. DOI: 10.1107/S1600536812015140/hb6707Isup3.cml

Additional supplementary materials:  crystallographic information; 3D view; checkCIF report

## Figures and Tables

**Fig. 1.**
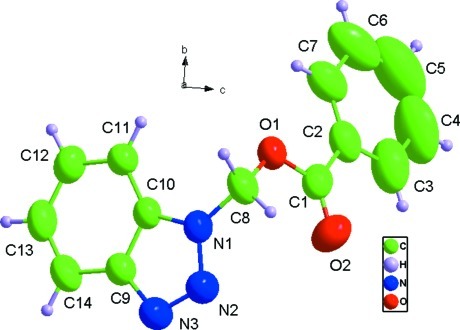
The molecular structure of the title compound with 50% probability displacement ellipsoids.

## supplementary crystallographic information

### Comment

Benzotriazole derivatives have been broadly researched due to their potential applications (Wan & Lv, 2010). Herein, we have synthesized a new benzotriazole derivative(Fig. 1), C_12_H_15_N_3_O_2_. Bond lengths and angles are comparable to other reported benzotriazol-1-yl intermediate derivatives (Zeng & Jian, 2009; Xu & Shen, 2012). Furthermore, the dihedral angle between the mean planes of the phenyl and benzotriazole rings is 76.80 (19)°. In the crystal, π–π stacking is observed between the inversion related phenyl rings of benzotriazolyl, the closest centroid-centroid distance being 3.754 (2)Å.

### Experimental

The title compound synthesis method is similar to that reported by Xu & Shen (2012), but methylene chloride was replaced by benzoyl chloride

### Refinement

The H atoms on the CH_2_ group were located by difference maps and freely refined without constraints. H atoms bonded to the remaining C atoms were included in calculated positions and treated as riding with C–H =0.93Å and *U*_iso_(H)=1.2Ueq(aromatic C).

### Crystal data

**Table:**

C_14_H_11_N_3_O_2_	*F*(000) = 528
*M**_r_* = 253.26	*D*_x_ = 1.303 Mg m^−^^3^
Monoclinic, *P*2_1_/*c*	Mo *K*α radiation, λ = 0.71073 Å
Hall symbol: -P 2ybc	Cell parameters from 1419 reflections
*a* = 10.7181 (4) Å	θ = 2.7–21.6°
*b* = 6.4826 (2) Å	µ = 0.09 mm^−^^1^
*c* = 18.7076 (7) Å	*T* = 296 K
β = 96.773 (3)°	Block, colorless
*V* = 1290.75 (8) Å^3^	0.20 × 0.20 × 0.18 mm
*Z* = 4	

### Data collection

**Table:**

Bruker SMART CCD diffractometer	2268 independent reflections
Radiation source: fine-focus sealed tube	1352 reflections with *I* > 2σ(*I*)
Graphite monochromator	*R*_int_ = 0.057
Detector resolution: 10.0 pixels mm^-1^	θ_max_ = 25.0°, θ_min_ = 2.2°
phi and ω scans	*h* = −12→12
Absorption correction: multi-scan (*SADABS*; Bruker, 2001)	*k* = −7→7
*T*_min_ = 0.982, *T*_max_ = 0.984	*l* = −22→22
9412 measured reflections	

### Refinement

**Table:**

Refinement on *F*^2^	Secondary atom site location: difference Fourier map
Least-squares matrix: full	Hydrogen site location: inferred from neighbouring sites
*R*[*F*^2^ > 2σ(*F*^2^)] = 0.060	H atoms treated by a mixture of independent and constrained refinement
*wR*(*F*^2^) = 0.162	*w* = 1/[σ^2^(*F*_o_^2^) + (0.0479*P*)^2^ + 0.4539*P*] where *P* = (*F*_o_^2^ + 2*F*_c_^2^)/3
*S* = 1.06	(Δ/σ)_max_ < 0.001
2268 reflections	Δρ_max_ = 0.14 e Å^−^^3^
181 parameters	Δρ_min_ = −0.17 e Å^−^^3^
0 restraints	Extinction correction: *SHELXL97* (Sheldrick, 2008), Fc^*^=kFc[1+0.001xFc^2^λ^3^/sin(2θ)]^-1/4^
Primary atom site location: structure-invariant direct methods	Extinction coefficient: 0.0073 (18)

### Special details

**Table:**

Geometry. All esds (except the esd in the dihedral angle between two l.s. planes) are estimated using the full covariance matrix. The cell esds are taken into account individually in the estimation of esds in distances, angles and torsion angles; correlations between esds in cell parameters are only used when they are defined by crystal symmetry. An approximate (isotropic) treatment of cell esds is used for estimating esds involving l.s. planes.
Refinement. Refinement of F^2^ against ALL reflections. The weighted R-factor wR and goodness of fit S are based on F^2^, conventional R-factors R are based on F, with F set to zero for negative F^2^. The threshold expression of F^2^ > 2sigma(F^2^) is used only for calculating R-factors(gt) etc. and is not relevant to the choice of reflections for refinement. R-factors based on F^2^ are statistically about twice as large as those based on F, and R- factors based on ALL data will be even larger.

### Fractional atomic coordinates and isotropic or equivalent isotropic displacement parameters (Å^2^)

**Table:**

	*x*	*y*	*z*	*U*_iso_*/*U*_eq_	
C1	0.7169 (3)	0.5849 (5)	0.27655 (16)	0.0599 (8)	
C2	0.8234 (3)	0.7177 (6)	0.30445 (18)	0.0672 (9)	
C3	0.9100 (4)	0.6382 (8)	0.3586 (2)	0.1029 (14)	
H3	0.8998	0.5062	0.3765	0.123*	
C4	1.0110 (5)	0.7578 (13)	0.3853 (3)	0.145 (2)	
H4	1.0696	0.7062	0.4215	0.174*	
C5	1.0258 (6)	0.9545 (14)	0.3586 (4)	0.157 (3)	
H5	1.0944	1.0342	0.3769	0.189*	
C6	0.9397 (5)	1.0320 (8)	0.3054 (3)	0.1179 (18)	
H6	0.9497	1.1645	0.2878	0.141*	
C7	0.8391 (3)	0.9146 (6)	0.2781 (2)	0.0814 (11)	
H7	0.7810	0.9671	0.2418	0.098*	
C8	0.5351 (3)	0.5700 (6)	0.19118 (18)	0.0614 (9)	
C9	0.6278 (3)	0.2459 (5)	0.05103 (16)	0.0564 (8)	
C10	0.6200 (3)	0.4440 (4)	0.07805 (14)	0.0505 (7)	
C11	0.6600 (3)	0.6164 (5)	0.04401 (16)	0.0608 (9)	
H11	0.6543	0.7485	0.0628	0.073*	
C12	0.7088 (3)	0.5800 (5)	−0.01930 (17)	0.0726 (10)	
H12	0.7380	0.6911	−0.0441	0.087*	
C13	0.7165 (3)	0.3815 (6)	−0.04810 (17)	0.0724 (10)	
H13	0.7504	0.3649	−0.0913	0.087*	
C14	0.6758 (3)	0.2130 (5)	−0.01465 (17)	0.0686 (9)	
H14	0.6796	0.0817	−0.0343	0.082*	
H1M	0.482 (3)	0.679 (5)	0.1671 (18)	0.078 (11)*	
H2M	0.491 (3)	0.493 (5)	0.2271 (18)	0.087 (11)*	
N1	0.5679 (2)	0.4166 (4)	0.14069 (12)	0.0571 (7)	
N2	0.5446 (3)	0.2141 (4)	0.15102 (14)	0.0729 (8)	
N3	0.5796 (3)	0.1092 (4)	0.09718 (15)	0.0752 (9)	
O1	0.64454 (19)	0.6783 (3)	0.22152 (10)	0.0584 (6)	
O2	0.6951 (2)	0.4154 (4)	0.29774 (13)	0.0830 (8)	

### Atomic displacement parameters (Å^2^)

**Table:**

	*U*^11^	*U*^22^	*U*^33^	*U*^12^	*U*^13^	*U*^23^
C1	0.065 (2)	0.067 (2)	0.0494 (17)	0.0088 (18)	0.0141 (16)	0.0005 (16)
C2	0.055 (2)	0.090 (3)	0.057 (2)	−0.0006 (19)	0.0115 (17)	−0.0160 (19)
C3	0.071 (3)	0.155 (4)	0.080 (3)	0.016 (3)	−0.003 (2)	−0.014 (3)
C4	0.075 (4)	0.240 (8)	0.114 (5)	0.002 (5)	−0.015 (3)	−0.043 (5)
C5	0.075 (4)	0.228 (8)	0.170 (7)	−0.039 (5)	0.018 (4)	−0.089 (7)
C6	0.085 (3)	0.125 (4)	0.152 (5)	−0.041 (3)	0.050 (3)	−0.057 (4)
C7	0.072 (3)	0.089 (3)	0.088 (3)	−0.018 (2)	0.030 (2)	−0.026 (2)
C8	0.061 (2)	0.070 (2)	0.0534 (19)	0.0043 (19)	0.0083 (18)	−0.0129 (18)
C9	0.064 (2)	0.0560 (17)	0.0497 (17)	0.0003 (15)	0.0079 (16)	−0.0034 (14)
C10	0.0551 (19)	0.0539 (17)	0.0423 (15)	0.0009 (14)	0.0045 (14)	−0.0016 (13)
C11	0.076 (2)	0.0547 (18)	0.0516 (17)	−0.0021 (16)	0.0078 (17)	−0.0012 (14)
C12	0.085 (3)	0.079 (2)	0.0553 (19)	−0.0057 (19)	0.0155 (19)	0.0080 (18)
C13	0.081 (3)	0.092 (3)	0.0465 (18)	0.006 (2)	0.0182 (17)	−0.0043 (18)
C14	0.081 (2)	0.070 (2)	0.0552 (19)	0.0090 (18)	0.0105 (18)	−0.0145 (17)
N1	0.0712 (18)	0.0564 (15)	0.0454 (13)	0.0007 (13)	0.0136 (13)	−0.0026 (12)
N2	0.103 (2)	0.0594 (17)	0.0583 (17)	−0.0114 (15)	0.0191 (16)	−0.0007 (14)
N3	0.110 (2)	0.0569 (16)	0.0611 (17)	−0.0071 (15)	0.0225 (17)	−0.0055 (14)
O1	0.0718 (15)	0.0583 (12)	0.0450 (11)	−0.0001 (11)	0.0064 (11)	−0.0033 (10)
O2	0.0905 (19)	0.0774 (16)	0.0804 (17)	0.0015 (13)	0.0077 (14)	0.0232 (13)

### Geometric parameters (Å, º)

**Table:**

C1—O2	1.200 (3)	C8—H1M	0.98 (3)
C1—O1	1.356 (3)	C8—H2M	1.00 (3)
C1—C2	1.476 (5)	C9—N3	1.379 (4)
C2—C7	1.385 (5)	C9—C10	1.386 (4)
C2—C3	1.389 (5)	C9—C14	1.404 (4)
C3—C4	1.377 (7)	C10—N1	1.367 (3)
C3—H3	0.9300	C10—C11	1.380 (4)
C4—C5	1.385 (9)	C11—C12	1.371 (4)
C4—H4	0.9300	C11—H11	0.9300
C5—C6	1.371 (8)	C12—C13	1.401 (4)
C5—H5	0.9300	C12—H12	0.9300
C6—C7	1.369 (6)	C13—C14	1.357 (4)
C6—H6	0.9300	C13—H13	0.9300
C7—H7	0.9300	C14—H14	0.9300
C8—O1	1.426 (4)	N1—N2	1.355 (3)
C8—N1	1.443 (4)	N2—N3	1.306 (3)
			
O2—C1—O1	122.9 (3)	H1M—C8—H2M	112 (3)
O2—C1—C2	126.1 (3)	N3—C9—C10	108.9 (3)
O1—C1—C2	111.0 (3)	N3—C9—C14	130.8 (3)
C7—C2—C3	120.2 (4)	C10—C9—C14	120.3 (3)
C7—C2—C1	122.2 (3)	N1—C10—C11	133.0 (3)
C3—C2—C1	117.6 (4)	N1—C10—C9	103.9 (2)
C4—C3—C2	119.0 (5)	C11—C10—C9	123.1 (3)
C4—C3—H3	120.5	C12—C11—C10	115.5 (3)
C2—C3—H3	120.5	C12—C11—H11	122.3
C3—C4—C5	120.4 (7)	C10—C11—H11	122.3
C3—C4—H4	119.8	C11—C12—C13	122.4 (3)
C5—C4—H4	119.8	C11—C12—H12	118.8
C6—C5—C4	120.2 (7)	C13—C12—H12	118.8
C6—C5—H5	119.9	C14—C13—C12	121.7 (3)
C4—C5—H5	119.9	C14—C13—H13	119.1
C7—C6—C5	120.0 (6)	C12—C13—H13	119.1
C7—C6—H6	120.0	C13—C14—C9	116.9 (3)
C5—C6—H6	120.0	C13—C14—H14	121.6
C6—C7—C2	120.2 (5)	C9—C14—H14	121.6
C6—C7—H7	119.9	N2—N1—C10	110.4 (2)
C2—C7—H7	119.9	N2—N1—C8	120.7 (3)
O1—C8—N1	110.3 (3)	C10—N1—C8	128.8 (3)
O1—C8—H1M	103.6 (18)	N3—N2—N1	108.6 (2)
N1—C8—H1M	111.5 (19)	N2—N3—C9	108.1 (2)
O1—C8—H2M	114.3 (19)	C1—O1—C8	116.9 (3)
N1—C8—H2M	105.7 (19)		
			
O2—C1—C2—C7	−177.9 (3)	C11—C12—C13—C14	−0.1 (6)
O1—C1—C2—C7	3.0 (4)	C12—C13—C14—C9	−1.2 (5)
O2—C1—C2—C3	2.5 (5)	N3—C9—C14—C13	179.8 (3)
O1—C1—C2—C3	−176.6 (3)	C10—C9—C14—C13	1.9 (5)
C7—C2—C3—C4	−0.1 (6)	C11—C10—N1—N2	−180.0 (3)
C1—C2—C3—C4	179.5 (4)	C9—C10—N1—N2	−0.4 (3)
C2—C3—C4—C5	0.1 (8)	C11—C10—N1—C8	1.9 (6)
C3—C4—C5—C6	0.1 (10)	C9—C10—N1—C8	−178.5 (3)
C4—C5—C6—C7	−0.4 (9)	O1—C8—N1—N2	119.7 (3)
C5—C6—C7—C2	0.4 (6)	O1—C8—N1—C10	−62.3 (4)
C3—C2—C7—C6	−0.1 (5)	C10—N1—N2—N3	−0.1 (4)
C1—C2—C7—C6	−179.7 (3)	C8—N1—N2—N3	178.3 (3)
N3—C9—C10—N1	0.7 (3)	N1—N2—N3—C9	0.5 (4)
C14—C9—C10—N1	179.0 (3)	C10—C9—N3—N2	−0.8 (4)
N3—C9—C10—C11	−179.7 (3)	C14—C9—N3—N2	−178.9 (3)
C14—C9—C10—C11	−1.3 (5)	O2—C1—O1—C8	3.2 (4)
N1—C10—C11—C12	179.6 (3)	C2—C1—O1—C8	−177.7 (2)
C9—C10—C11—C12	0.0 (5)	N1—C8—O1—C1	−81.1 (3)
C10—C11—C12—C13	0.7 (5)		
